# Human nasal microbiota shifts in healthy and chronic respiratory disease conditions

**DOI:** 10.1186/s12866-024-03294-5

**Published:** 2024-04-27

**Authors:** Aleksandras Konovalovas, Julija Armalytė, Laurita Klimkaitė, Tomas Liveikis, Brigita Jonaitytė, Edvardas Danila, Daiva Bironaitė, Diana Mieliauskaitė, Edvardas Bagdonas, Rūta Aldonytė

**Affiliations:** 1 Life Sciences Center, Institute of Biosciences, Vilnius University, Vilnius, Lithuania; 2 Clinic of Chest Diseases, Immunology, and Allergology, Faculty of Medicine, Vilnius University, Vilnius, Lithuania; 3 Centre of Pulmonology and Allergology, Vilnius University Hospital Santaros Klinikos, Vilnius, Lithuania; 4 State Research Institute Centre for Innovative Medicine, Vilnius, Lithuania

**Keywords:** Upper respiratory tract, Nasal microbiota, Biomarkers

## Abstract

**Background:**

An increasing number of studies investigate various human microbiotas and their roles in the development of diseases, maintenance of health states, and balanced signaling towards the brain. Current data demonstrate that the nasal microbiota contains a unique and highly variable array of commensal bacteria and opportunistic pathogens. However, we need to understand how to harness current knowledge, enrich nasal microbiota with beneficial microorganisms, and prevent pathogenic developments.

**Results:**

In this study, we have obtained nasal, nasopharyngeal, and bronchoalveolar lavage fluid samples from healthy volunteers and patients suffering from chronic respiratory tract diseases for full-length 16 S rRNA sequencing analysis using Oxford Nanopore Technologies. Demographic and clinical data were collected simultaneously. The microbiome analysis of 97 people from Lithuania suffering from chronic inflammatory respiratory tract disease and healthy volunteers revealed that the human nasal microbiome represents the microbiome of the upper airways well.

**Conclusions:**

The nasal microbiota of patients was enriched with opportunistic pathogens, which could be used as indicators of respiratory tract conditions. In addition, we observed that a healthy human nasal microbiome contained several plant- and bee-associated species, suggesting the possibility of enriching human nasal microbiota via such exposures when needed. These candidate probiotics should be investigated for their modulating effects on airway and lung epithelia, immunogenic properties, neurotransmitter content, and roles in maintaining respiratory health and nose-brain interrelationships.

**Supplementary Information:**

The online version contains supplementary material available at 10.1186/s12866-024-03294-5.

## Background

An increased interest in nasal microbiota arises from its role in respiratory health, its proximity to the brain, possible implications in neurodegenerative diseases, and the uninvestigated diagnostic and therapeutic potential it harbors. A lot of the microbiota-centered research still focuses on the gut-brain axis, however, the nose-brain axis is so powerful and swift that its role in modulating the immune system, local respiratory homeostasis, and ultimately, its influence over the nervous system cannot be ignored any longer.

The nasal microbial communities are significantly less abundant than the ones in the gut but nonetheless diverse and understudied. A growing body of evidence suggests that nasal microbiota can influence the physiology and pathophysiology of the central nervous system [1]. It was demonstrated in laboratory animals and in post-mortem tissues that nasally located pathogens can enter through the olfactory epithelium cribriform plate and travel via the olfactory tract, reaching and infecting the brain neurons [2, 3]. Therefore, the studies of microbiome composition and its interaction with host immune and nervous systems are highly anticipated.

The human nose houses a complex bacterial community, where bacteria belonging to the phyla *Actinobacteria, Firmicutes* and *Proteobacteria* dominate, while *Fusobacteria* and *Bacteroidetes* are present in significantly lower amounts [4–6]. The most common genera in the healthy nasal cavities have been previously reported as *Corynebacterium, Propionibacterium, Streptococcus, Staphylococcus, Moraxella, Haemophilus* [7, 8]. However, the composition and diversity of this community is very dynamic and varies under the influence of many factors, e.g. age, diseases, smoking status, medications used, environmental factors, and other microbiotas [7]. Nasal microbiota is impacted by our lifestyle factors, like air pollution and exposure to greenery [9], the widespread use of antibiotics [10], intranasal drugs and medications [11], allergens [12], indoor pollution [13] tobacco smoking and vaping [14, 15]. Human nasal microbiota needs constant replenishment from other microbiotas (oral, gut) and, importantly, the ambient aerobiome, which are also influenced by anthropogenic factors, further complicating the overall “healthy microbiota” picture.

In chronic respiratory tract diseases, such as bronchiectasis (BE), the microbial factor and dysfunctional immunity contribute to the pathophysiology, while the underlying cause of the disease is still unclear in a large part of cases [16]. While the lower respiratory tract microbiota of BE patients has been explored to some extent [17–21], the content and role of nasal microbiota in BE patients is still sparse [22]. However, in similar morbidities, like chronic obstructive pulmonary disease, lung microbiota closely reflected the oral, and nasal microbiota [23].

To understand the relationship of the upper respiratory tract microbiota composition and suggest its possible implications for the induction of the pathogenicity in the respiratory tract diseases we have analyzed the upper respiratory tract microbiota composition of modern Lithuanians. Our aim was to set a preliminary picture of a healthy nasal microbiome in comparison to a diseased one and to select possible biomarkers for a less-invasive lower airway health condition detection.

## Methods

### Study design and sample collection

The study has been approved by the Lithuanian Bioethics Committee (approval #2021/2-1308-786) in accordance with the current guidelines and regulations. Informed consent was obtained from all the participants. The specimens from the nasal and nasopharyngeal cavities were collected from a diverse cohort of individuals, consisting of healthy volunteers and patients with diagnosed respiratory tract diseases (the majority of patients were diagnosed with bronchiectasis). For BE patients, bronchoalveolar lavage fluid/bronchial aspirate was also collected and its microbial composition analyzed in another study (Konovalovas et al., 2023 manuscript submitted for publication). Main individual person data is presented in Supplementary Table 1, including information about owning a furry pet and working in an aromatic substance-rich environment. Nasal and nasopharyngeal swabs were collected as described elsewhere [13] and immersed into DNA/RNA Shield (Zymo Research) immediately after collection and kept at + 4℃ until the DNA extraction step.

### DNA purification and sequencing

DNA extraction was performed using Zymo Research Quick-DNA™ Microprep Plus Kit (Biological Fluids & Cells protocol) according to the manufacturer’s recommendations.

The sequencing library for 16 S rRNA was prepared using the SQK-RAB204 or SQK-16S024 rapid 16 S amplicon barcoding kits by Oxford Nanopore Technologies (ONT) adhering to the guidelines provided by the manufacturer. The entire 16 S rRNA gene was amplified from each sample using 10 ng/uL of total DNA. This amplification was executed using LongAmp® Taq polymerase master mix (New England Biolabs), and the ONT-supplied barcoded primer pair (27 F and 1492R). The PCR process followed a specific temperature cycling program: it began with a 1-minute denaturation at 95 °C, proceeded through 35 cycles: 20 s at 95 °C, 30 s at 55 °C, and 1 min at 65 °C, and concluded with a final extension phase lasting 5 min at 65 °C. According to ONT instructions, the barcoded amplicons were purified using AMPure XP beads (Beckman Coulter). Sample DNA concentration was determined using the NanoDrop One spectrophotometer (Thermo Fisher Scientific) and pooled up to 10 samples in equimolar concentrations for 100 pmol. The library was loaded onto the R9.4.1 Flongle flowcell and sequenced for 24 h using a MinION with Flongle adapter (ONT). All sequencing data are publicly available in the European Nucleotide Archive (https://www.ebi.ac.uk/ena) under accession numbers PRJEB70318 and PRJEB70777.

### Full-length 16 S rRNA sequencing analysis

The raw fast5 files underwent base-calling with Guppy (version 6.5.7 + ca6d6af) (Guppy basecalling software https://community.nanoporetech.com (2023)) and minimap2 [24] (version 2.24-r1122), producing fastq files. Default settings were maintained, except for the use of the dna_r9.4.1_450bps_sup model, which was chosen to enhance base-calling precision, and excluding reads with a mean q-score below 8. The reads in the fastq files, generated from the base-calling process, were demultiplexed and adapters trimmed using Porechop (https://github.com/rrwick/Porechop) (version 0.2.4). The taxonomic annotation and relative abundance/count data for the basecalled and demultiplexed fastq files were determined using the Emu [25] software (version v3.4.4), employing its default settings and database for the analysis.

Additionally, we employed the complement of the ThetaYC index (1 - ThetaYC dissimilarity index) to compare differences in community structure [26] across various respiratory sites, including the nose, nasopharynx, and lungs. This index ranges from 0 to 1, with higher values indicating greater similarity in microbial communities across different sites. A value of 1 suggests highly similar communities, while a value of 0 indicates no similarity.

We performed cluster analyses of the nasal microbiome using two beta diversity indices: the weighted UniFrac method [27] and the Ochiai index (also known as Cosine similarity index). The weighted UniFrac, facilitated by the phyloseq [28] package (version 1.42.0), emphasizes species’ relative abundance, whereas the Ochiai index focuses solely on the presence of species. Before analysis, we excluded all taxa with less than 0.1% relative abundance in the sample.

Hierarchical clustering was performed using the Ward.D2 method, as outlined by Murtagh and Legendre [29]. Optimal cluster numbers were determined using silhouette and elbow score methods. Statistical comparisons between groups were executed using the Kruskal-Wallis Rank Sum Test, with pairwise group comparisons via the Pairwise Wilcoxon Rank Sum Test. P-values for both tests were adjusted using the Benjamini & Hochberg method. All analyses were conducted in R version 4.2.2.

## Results

### Demographic and clinical characteristics of participants

In this study, specimens from the upper airways (nasal cavity and nasopharynx) were collected from a diverse cohort of 100 individuals. 3 samples did not yield microbiota composition data of adequate quality and thus were removed from the analysis. Of the remaining 97 participants, 43 participated as healthy volunteers (no diagnosed respiratory tract diseases at the moment of sample collection). 30 of the healthy participants were furry pet owners (dogs, cats, or both), 11 had no pets, for two healthy participants information regarding pet ownership was unknown. We have chosen to include pet ownership as a factor in this study due to the reported CHILD study and other results on the beneficial enrichment of intestinal microbiomes in those who own furry pets [30]. In addition, 11 healthy volunteers were workers at the herbal distillery where plant-derived volatile organic compounds (VOCs) remain in high numbers for the entire working day and contain major volatile plant-derived compounds linalool, eucalyptol, d-limonene, ρ-cymene, terpinene-4-ol and others [31]. In addition to VOC, plant material also delivers diverse air microbiomes [32]. Moreover, plant-derived volatile aromatic compounds are already known modulators of gut microbiome in laboratory animals [33–35]. This group of study participants was chosen based on the hypothesis that an atmosphere enriched with plant-derived VOCs and plant-derived microorganisms may be an important player in human nasal microbiome modulation.

54 study participants were suffering from chronic inflammatory respiratory tract diseases: 47 were diagnosed with BE at the time of sampling, and 7 had other chronic inflammatory lung conditions, undiagnosed at the time of sampling; the available metadata is presented in Supplementary Table 1.

The average age of study participants was 55 years (SD = 16.5), where healthy volunteers’ mean age was 43.4 years (SD = 13.6), and patients’ mean age was 64.3 (SD = 12.4) years. The gender representation included 69 females (71.1%) in total; with 33 (34%) females among the healthy volunteers and 36 (37.1%) females among patients.

### Comparative analysis of nasal, nasopharyngeal, and lower respiratory tract microbiomes in bronchiectasis patients

We aimed to investigate the potential of using upper respiratory airway microbiome composition as a biomarker for lower respiratory airway disorders. The focus on the upper respiratory tract was due to the less invasive nature of sample collection, which could facilitate its intended use as a diagnostic tool. To compare upper and lower respiratory tract microbiota, nasal and nasopharyngeal swabbing samples were collected representing the upper respiratory tract, and bronchoalveolar lavage fluid or bronchial aspirate were collected via bronchoscopy, representing the lower one. We selected a clinical cohort of patients diagnosed with BE, in the clinically stable phase at the time of sample collection. Out of 47 BE patients, 46 yielded nasal, nasopharyngeal swabbing samples and bronchoalveolar lavage fluid or bronchial aspirate samples of appropriate quality and were used in the analysis. Two healthy volunteers agreed to undergo bronchoscopy and their upper and lower respiratory tract samples were also included in the analysis. For the rest of the healthy volunteers used in further comparative analysis only the upper airway microbiomes were analysed.

**Fig. 1:**
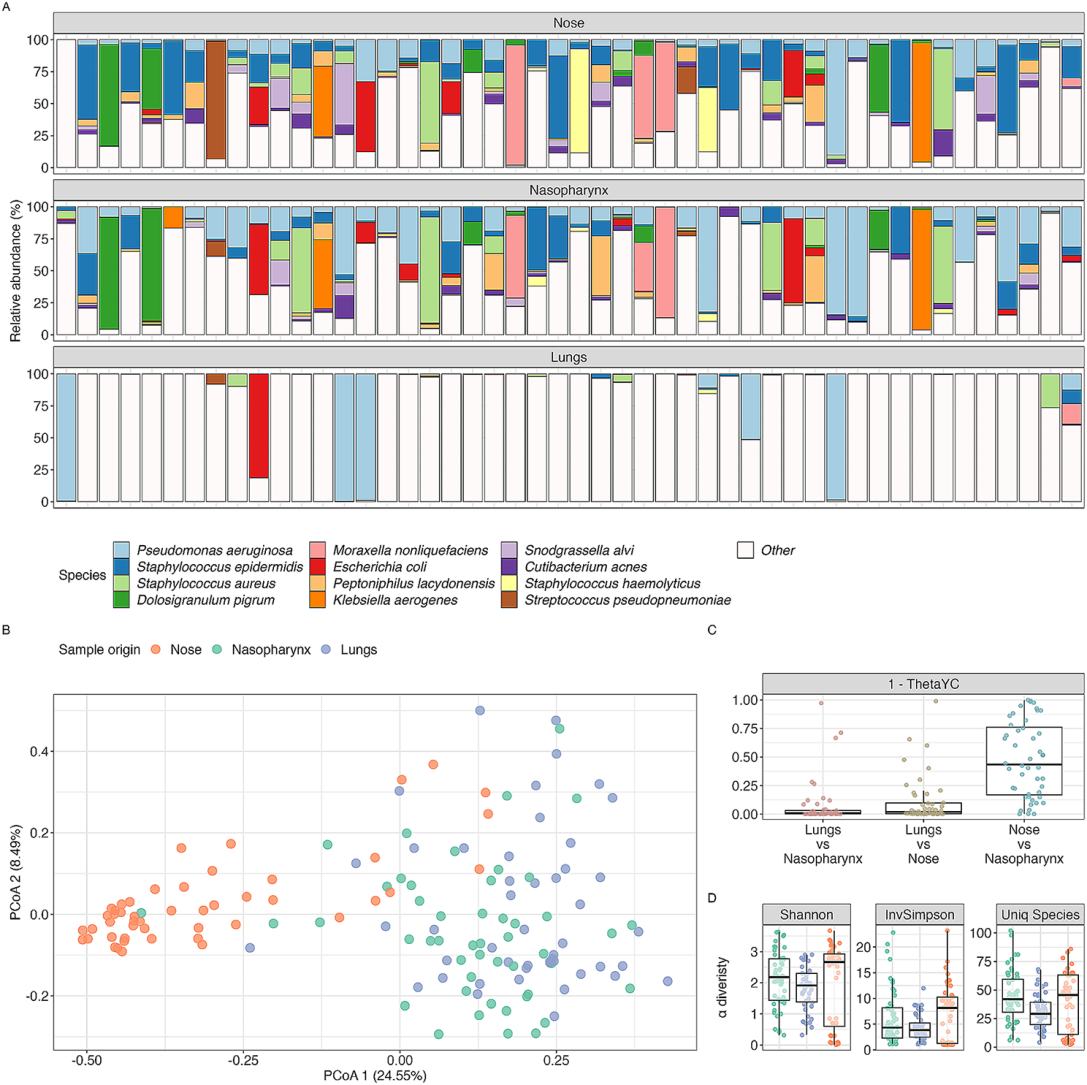
Distribution and diversity of microbial species in different respiratory tract sites. **A**) The relative abundance of microbial species in the nose, nasopharynx, and lungs. Each color represents a different species, with the key below indicating the specific organisms. **B**) The principal coordinate analysis (PCoA) based on weighted UniFrac distances, comparing the microbial communities from the nose (orange), nasopharynx (blue), and lungs (green). **C**) The beta-diversity comparison across the three respiratory sites uses the complement of the ThetaYC index (1 - ThetaYC dissimilarity index), where individual data points represent distinct samples.​​ Higher values of this index denote higher similarity in the microbial communities across different respiratory sites. **D**) Boxplots of alpha-diversity metrics: Shannon index, Inverted Simpson index, and species richness across the sampled sites. These visualizations collectively suggest distinct microbial profiles and diversity within the upper and lower respiratory tracts, with nasal swabs demonstrating higher species richness and diversity indices, making them potential non-invasive diagnostic biomarkers for lower respiratory tract disorders

A comprehensive analysis of the lung microbiota in BE patients was conducted and is presented in another of our studies (Konovalovas et al., 2023, manuscript submitted for publication). Our first interest in this study was comparing the lung microbiota profiles with nasal and nasopharyngeal samples. Our findings indicated a similarity in the dominant species in both nasal and nasopharyngeal samples (Fig. 1A–B), however, we observed no significant correlation between the upper and lower respiratory tract microbiomes (Fig. 1A–C). The comparative evaluation of nasal and nasopharyngeal samples, as indicated by the complement of the ThetaYC index (Fig. 1C), confirmed a marked similarity in both of the upper respiratory tract microbiota compositions. When comparing the richness and evenness between the nasopharynx and nasal microbiomes, we discovered that the nasal microbiome exhibited significant (*p* < 0.05) richness in the detected higher number of species (Fig. 1D). We also found that the Shannon index and Inverted Simpson index were more elevated in nasal swab samples than those from the nasopharynx (Fig. 1D). Additionally, nasal samples exhibited a significantly higher DNA concentration (data not shown), underscoring their better suitability for detailed analysis, thus we decided to use only nasal samples for the following assessments.

### Variations of nasal microbiomes across demographic and environmental factors in healthy individuals

To explore the utility of upper respiratory tract microbiome composition as an indicator for lower respiratory tract conditions, we examined whether variations in nasal microbiomes among healthy individuals could be attributed to demographic (sex) and environmental (pet ownership and intense plant-derived VOCs and plant-derived microorganism exposure) factors.

**Fig. 2:**
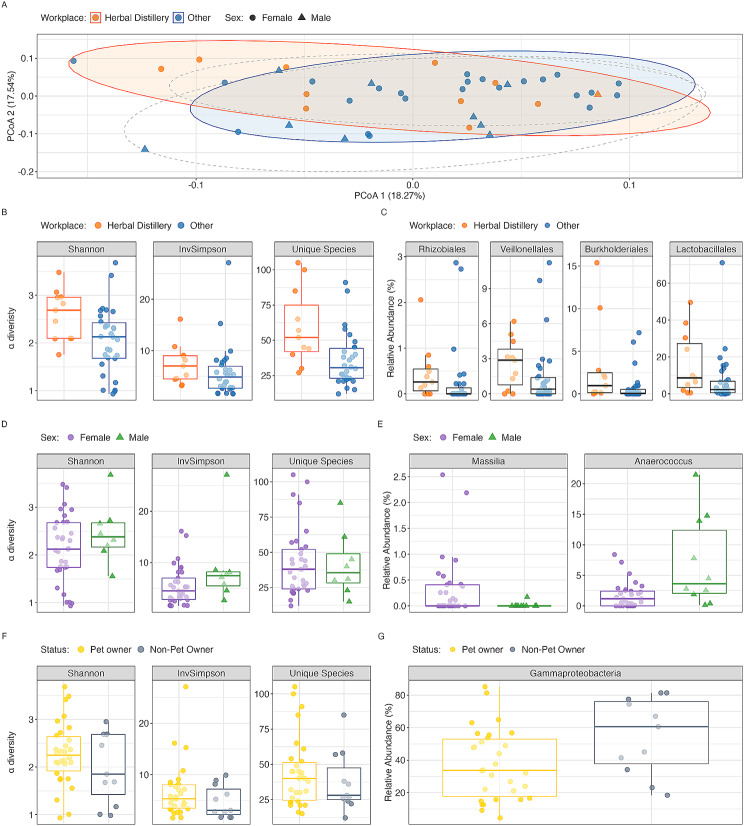
Comparative analysis of nasal microbiome diversity and abundance by sex or environmental factors in healthy individuals. **A**) A PCoA plot based on the weighted UniFrac distance, with orange dots indicating those from herbal distillery companies, and blue dots representing participants from other workplaces. Overlaid dashed ellipses categorize the groups based on sex, with triangles for male participants and circles for females, showing no distinct clustering within groups. **B**) Boxplots displaying alpha diversity indices: Shannon, Inverted Simpson, and Richness (number of unique identified taxonomies) across herbal distillery and other workplace groups. Participants from herbal distilleries exhibit statistically significant differences in all metrics (p-value < 0.05). **C**) Boxplots showing the relative abundance of statistically different (p-value < 0.05) order-level taxonomy organisms between the participants from herbal distilleries and other workplaces. **D**) Boxplots comparing alpha diversity indices between male and female participants, revealing no statistical differences. **E**) Boxplots illustrating genus-level organisms with statistically significant (*p* < 0.05) differences in relative abundance between different sex groups

Nasal microbiome composition analysis did not reveal significant differences in bacterial species at any taxonomic level between herbal distillery workers and other healthy volunteers (Supplementary Fig. 1), as well as other demographic and environmental factors analysed (Supplementary Fig. 2). Initial principal coordinate analysis (PCoA) utilizing weighted UniFrac distances revealed no distinct clustering by sex or environmental factors such as pet ownership and aromatic compound exposure (Fig. 2A). The PCoA plot demonstrates that these metadata categories do not lead to overt segregation within the microbiome data, suggesting a homogeneous distribution of microbial communities regardless of the investigated factors.

Despite no clear group segregation observed in PCoA analysis, we could detect minor differences between the groups. When the microbiome diversity was analyzed, the alpha diversity metrics highlighted that only aromatic compound exposure was associated with a significant increase in the Shannon index (p-value = 0.02) and richness (p-value = 0.005). The Inverted Simpson index presented only a trend toward significance (p-value = 0.07) (Fig. 2B). This could imply that exposure to diverse aromatic compounds may contribute to an equally diverse nasal microbiome, perhaps due to a broader spectrum of microbial substrates available in these environments. However, the observed trend in the Inverted Simpson index, without reaching statistical significance, might suggest that while the variety of species is greater, the dominance of particular species remains unaltered.

No significant differences were observed in alpha diversity measures between different sexes (Fig. 2D) or in pet ownership (Fig. 2F), or in combination with the workplace environment (Supplementary Fig. 3). The consistent alpha diversity across these groups suggests that, within the bounds of a healthy cohort, demographic and specific lifestyle factors may have limited influence on the nasal microbiome’s overall structure and diversity.

To verify the presence of specific organisms associated with these factors we conducted Wilcoxon rank-sum tests with Benjamini-Hochberg correction for multiple comparisons. This analysis revealed only one statistically significant difference in bacterial species at the class taxonomic level between pet owners and non-pet owners, particularly in *Gammaproteobacteria* (p-value = 0.034), showing a slight decrease in bacteria of this class in pet owners compared to non-pet owners (Fig. 2G).

However, distinct differences emerged when examining the factors of aromatic compound exposure and sex (Fig. 2C–E). Within the herbal distillery workers’ group, we observed an increase in the relative abundance of certain bacteria from the phyla *Proteobacteria* and *Firmicutes*. Specifically, bacteria from the *Burkholderiales* order were more prevalent (p-value = 0.024), with an average relative abundance four times higher in the herbal distillery workers’ group than in healthy controls. Notably, bacteria of this order were detected in almost all individuals (10 out of 11) in the herbal distillery workers’ group, in contrast to only 50% in the other healthy volunteers group. Another statistically significant finding (p-value = 0.01) was the higher average abundance of bacteria from the *Rhizobiales* order in the herbal distillery workers’ group, where 8 out of 11 individuals harbored this bacteria compared to only 28% in the other healthy volunteers group.

Additionally, bacteria from the *Lactobacillales* order within the *Firmicutes* phylum were found to be increased by an average relative abundance fold of 3.8 in the herbal distillery workers’ group. These bacteria were present in all individuals exposed to herbal distillery environment and nearly all other healthy volunteers. Moreover, bacteria from the *Veillonellales* order, also part of the *Firmicutes* phylum, showed an average relative abundance increase of more than sevenfold in the herbal distillery workers’ group. These bacteria were found in 82% of the individuals in the herbal distillery workers’ group compared to 66% of the other healthy volunteers’ group.

Continuing our analysis of the influence of sex on nasal microbiome composition, we discovered several statistically significant differences at the genus level between females and males. While most bacteria showing variance were either in low abundance or relatively rare within the groups, two exceptions stood out: bacteria from the *Massilia* genus and those from the *Anaerococcus* genus (Fig. 2E).

Bacteria from the *Massilia* genus (p-value = 0.03) were found to be specific to females. The identification of *Massilia* in this demographic is notable, given its rarity or complete absence in male participants. On the other hand, bacteria from the *Anaerococcus* genus (p-value = 0.008) were predominantly associated with males, exhibiting an average abundance that was approximately four times higher than in females.

### Nasal microbiome composition in health and disease

When the nasal microbiomes of all the study participants (clinical cohort and healthy volunteers) was analysed, in the nasal microbiota of 878 different bacterial species were detected. However, only half of them (404 species) were found in 2 or more individuals. Only 43 species were common for > 20%, and only 15 species were common for more than 50% of study participants, indicating that nose microbiota is very variable among people. Since the species variation was very high, we aimed to analyze only the presence or absence of certain species and other taxa in the microbiota, not taking the relative abundance of each taxon into account, as our aim was to detect potential biomarkers that could indicate pathology-related changes in the lower respiratory tract.

When we compared the microbiota composition of healthy participants and participants suffering from respiratory tract diseases, two bacterial species, *Pseudomonas aeruginosa* and *Staphylococcus epidermidis*, were found in most of the samples (> 80% of both studied groups). Another commonly found bacterium, *Cutibacterium acnes*, was also common for both groups, however, it was slightly less common in the patient group (95% of healthy volunteers had *C. acnes* as opposed to 81% of patients). When we looked into which species were less common in the patients’ group as opposed to healthy volunteers, another bacterium from phylum *Actinobacteria, Corynebacterium accolens*, was more often found in healthy nasal microbiota as well as *Firmicutes* representatives from *Peptoniphilaceae* family (*Anaerococcus octavius, Anaerococcus urinomassiliensis, Peptoniphilus lacydonensis, Peptoniphilus grossensis*) and phylum *Proteobacteria* representative from *Neisseriaceae* family *Snodgrassella alvi*. In the majority (> 50%) of the patients’ nose microbiota the most noticeable increase of several *Proteobacteria* species(*Stenotrophomonas maltophilia, Delftia acidovorans* and *Mesorhizobium* sp.) was observed, only 7–14% of healthy microbiota possessing these bacteria, while over 50% of the patients’ microbiota did. Some phylum *Firmicutes* representatives were also observed in higher prevalence, such as *Streptococcus mitis, Streptococcus oralis* and *Veillonella dispar*.

### Clustering nasal microbiomes for respiratory biomarkers

**Fig. 3:**
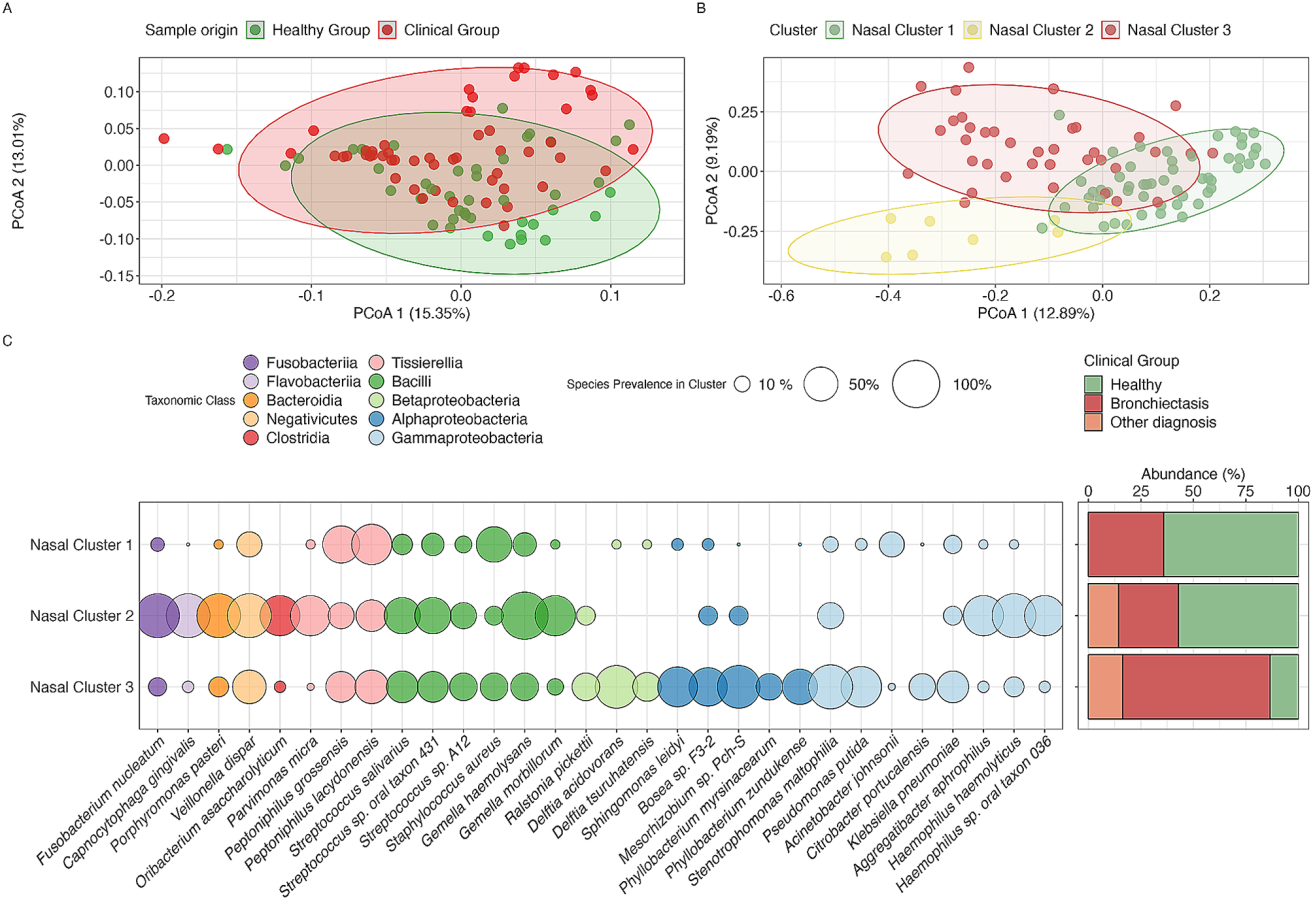
Comprehensive Analysis of Microbiome in Respiratory Health and Disease. **A**) PCoA based on the weighted UniFrac distance, highlighting the relative abundance of species. The green dots represent healthy individuals, while the red dots denote the clinical group, which includes BE patients and others with chronic inflammatory lower airway conditions. The ellipses encase clusters within each group, showcasing the microbiome variability between health and disease states. **B**) PCoA based on the Ochiai distance, focusing solely on the presence metrics of the species. The data points are colored by clusters identified via hierarchical clustering based on Ochiai distance. Each cluster is distinctly colored to illustrate the microbiome composition differences discerned through this method. **C**) The bubble plot of the most prevalent species differentiating the clusters, with the circle size representing the percentage of individuals harboring a specific species. The colors correspond to the taxonomic class of each species. Accompanying this plot is a bar chart displaying the abundance of individuals from each clinical group within the clusters, providing insights into the species distribution across different health conditions

To identify potential biomarkers associated with respiratory system diseases, we conducted a series of clustering analyses employing different beta diversity indices. Our initial analysis with the weighted UniFrac distance did not yield a specific separation of clinical groups in the nasal microbiome (Fig. 3A). Therefore, we shifted our focus to the Ochiai distance, which prioritizes the presence of species rather than their abundance. Utilizing the Ochiai distance led to a better separation between the groups. Hierarchical clustering based on this distance allowed us to delineate three distinct clusters (Fig. 3B). The first and the third casal clusters assembled the majority of the samples. Although all three clusters contained both healthy volunteer and patient samples, some tendencies could be observed: in Nasal Cluster 1, containing 53 samples, the majority of samples belonged to healthy volunteers (34 samples, or 64%), while in Nasal Cluster 3, containing 37 samples, a clear majority belonged to patients (32 samples, or 86.5%). The Nasal cluster 2 contained only 7 samples, and they were equal parts belonging to healthy volunteers and patients (4 and 3 samples, respectively).

Since Nasal Cluster 1 had more samples from healthy volunteers and Nasal Cluster 3 was mostly patients’ microbiota samples, however, they both still contained both, we analyzed the metadata available to find any differences in the patients attributed to both clusters. The only noticeable difference between the patients in Nasal Clusters 1 and 3 was the disease duration - the patients from Cluster 1 have had a longer diagnosed disease (average was 11.8 years; median − 6 years) than the ones from Cluster 3 (average was 4.6 years; median was 1 year).

When we analyzed the microbiota composition between Nasal Clusters 1 and 3, similarities and differences in the presence of bacteria belonging to different phyla could be observed (Fig. 3C). The *Firmicutes* representatives (belonging to classes *Bacilli, Tissierellia, Negativicutes*) had no clear differences between the clusters, apart from *V. dispar*, which as mentioned earlier, had a higher prevalence in the patients’ microbiomes; a similar trend was observed in Cluster 3 samples. More pronounced differences could be observed in phylum *Proteobacteria*, where representatives from several classes had distinct profiles in Nasal Clusters 1 and 3. Several bacterial species from *Gammaproteobacteria* class were more common in Nasal Cluster 3. Although *P. aeruginosa* was equally common in both Nasal Clusters 1 and 3, another *Pseudomonas* species, *P. putida*, was mostly found in cluster 3. A similar pattern was observed for another opportunistic pathogen of *Gammaproteobacteria* class, *Stenotrophomonas maltophilia*, as well as *Delftia acidovorans* belonging to class *Betaproteobacteria*. Class *Alphaproteobateria* also had several representatives that were significantly more common in Cluster 3 (*Bosea* sp. F3-2, *Mesorhizobium* sp. Pch-S, *Sphingomonas leidyi*). Phylum *Baicteroidetes* had a few bacterial species (*Porphyromonas pasteri, Capnocytophaga gingivalis*) that were more common in cluster 3; however, the mentioned bacteria were found in a very limited number of microbiomes.

## Discussion

When comparing a cohort of BE patients who donated nasal, nasopharyngeal, and BAL samples for this study, we have found that nasal and nasopharyngeal microbiomes are very closely correlated. These findings open the possibility of relying on nasal samples when planning a broad, self-collecting testing campaign in case of a pandemic or similar situation. It was already shown that self-collected and staff-collected samples function equally well [36].

As our study confirmed, the upper airway (nose and nasopharynx) does not accurately represent the microbiome composition of the lower airways and lungs, which has been previously observed in other studies [37]. However, the upper airways can be considered a potential microbial source for the lower ones [38]. Therefore, for the direct diagnostic assessment of the lower respiratory tract, one should use induced sputum, bronchoscopy, etc. On the other hand, nasal samples are sufficient for the evaluation of the microbiome of the upper airways. This aligns with the results of other studies and adds needed information on the feasibility of nasal microbiome tests [39].

No differences in higher taxa composition were observed between healthy volunteers and patient groups, indicating that nasal microbiota is not greatly impacted by changes observed in the lower respiratory tract. However, our search for biomarkers showed several lower taxa that could be used as indicators.

Interestingly, *P. aeruginosa* was found in more than 90% of cases in our study, including both healthy and patients’ groups, which was higher frequency than expected [40]. However, an increase in *P. aeruginosa* instances has been previously detected in the upper respiratory tract of people after viral infections: several studies analyzing microbiota composition after SARS-CoV-2 and other respiratory tract virus infections reported an increased abundance of opportunistic pathogens, especially *P. aeruginosa* [41–44]. Since the samples in our study were collected during or shortly after the COVID-19 pandemic, the changes in the microbiota and increase of *P. aeruginosa* incidences could be in part attributed to participants encountering the respiratory virus.

Apart from similarities, differences in microbiota composition were found in the healthy volunteers group as well as the patients’ group. For instance, our study participants, working in the herbal distillery (a part of the healthy volunteer group), have displayed a distinctive group of microbiota composition. Aromatic (airborne) and herbal compounds in the working environment were associated with *Burkholderia* genus increase in the nasal microbiomes of healthy study participants working at a herbal distillery suggesting possibly friendly species of this diverse and heterogeneous genus, representing soil and water residents, plant-associated bacteria, and human pathogens (especially cystic fibrosis cases). Interestingly, it was demonstrated that the symbiotic, plant-based *Burkholderia* species are not pathogenic to mammals based on both functional and genomic data [45]. It is an important observation since the many *Burkholderia* strains, e.g., endophytic or nitrogen-fixing, show great promise as agents for plant growth promotion, bioremediation and biotechnologies. Thus, the search for human health-friendly *Burkholderia* strains is ongoing [46].

Despite the differences in the healthy group, several bacteria, which could be considered as candidates for beneficial microorganisms, were more commonly identified in healthy and less common in BE patients’ nasal microbiomes, for instance, *Corynebacterium accolens*, *Anaerococcus octavius, Anaerococcus urinomassiliensis, Peptoniphilus lacydonensis, Peptoniphilus grossensis* and *Snodgrassella alvi.* Some of these bacteria are already known players of healthy microbiota and their protective properties have been previously described [47–49]. However, additional research into each of them should be carried out, since some of them also have been observed to exhibit pathogenic properties in certain situations [50, 51]. Interestingly, *Snodgrassella alvi* has been known in other contexts, as a bee gut health indicator [52]. It is tempting to speculate that enrichment of *S. alvi* may add a probiotic effect on human nasal microbiota as well as in bees. Moreover, a recent study shows that *S. alvi* modulates tryptophan metabolism in the gut of bees engaging in gut-brain crosstalk [53]. This notion supports the idea of psychobiotic activities of *S. alvi* and stimulates future research into the abilities of this bacteria to function within the nose-brain axis in humans.

The patients with respiratory tract disorders also showed indicator organisms in our study. Compared to healthy volunteers, the patients’ microbiota more commonly included representatives of class *Gammaproteobacteria*, and often they were already described previously as opportunistic pathogens. In the majority of the patients’ nose microbiomes *S. maltophilia* and *D. acidovorans* were detected, while only a few healthy volunteers had them in their microbiota. Despite being a ubiquitous environmental microorganism [54], *S. maltophilia* has recently come to light as a potent opportunistic pathogen, causing difficult-to-treat infection in immunocompromised patients due to its innate antibiotic resistance [55]. *D. acidovorans* is generally considered a nonpathogenic environmental microorganism, however, it has been demonstrated to be linked to lung disorders and other infections in patients who are immunosuppressed or immunocompromised [56–58]. Similarly, *P. putida*, despite being mostly described as a saprophytic organism, has been known to cause opportunistic infections [59, 60]. The presence of opportunistic pathogens in the nasal microbiota, especially the innately antibiotic-resistant *S. maltophilia*, could be a risk indication for an opportunistic infection for patients with chronic lower respiratory tract diseases. Other bacteria, which were more common in patients’ microbiota (*Bosea* sp. F3-2, *Mesorhizobium* sp. Pch-S, *Sphingomonas leidyi*) have not been described as able to cause infections, and their involvement in pathogenesis or their role as biomarkers have to be elucidated. However, other bacteria from the same genus have, indicating their potential to become opportunistic pathogens [61–63].

Overall, our results suggest that the nose microbiome in a healthy person may harbor specific markers, as can the microbiome of people with chronic lower respiratory tract diseases. Nasal microbiota can become a resource of diagnostic biomarkers and serve as a therapeutic tool to limit certain pathogens or deliver messages via the nasal-brain axis.

## Conclusions

- The human nasal microbiome can be used as a representative of the upper airways. However, there was no correlation between the nose and the lower airway microbiome composition in our study.
- Healthy human nasal microbiome harbored several delegates of plant-associated microbiotas suggesting the possibility of enriching human nasal microbiota via such exposures; certain strains from healthy human nasal microbiomes may be regarded as future probiotics applicable in the respiratory tract.
- The nasal microbiota of chronic lower respiratory tract disease patients was enriched by opportunistic pathogens, mostly belonging to phylum *Proteobacteria*.

### Electronic supplementary material

Below is the link to the electronic supplementary material.

Supplementary Material 1

Supplementary Material 2

## Data Availability

All sequencing data are publicly available in the European Nucleotide Archive (https://www.ebi.ac.uk/ena) under accession numbers PRJEB70318 and PRJEB70777.
