# Supplementary material for: Human nasal microbiota shifts in healthy and chronic respiratory disease conditions
Source: BMC Microbiol. 2024 Apr 27;24:150. doi: 10.1186/s12866-024-03294-5 (PMC11055347; doi:10.1186/s12866-024-03294-5)
Supplement: Supplementary file 2 — Supplementary Material 2 [file 12866_2024_3294_MOESM2_ESM.pdf]

Supplementary Figures

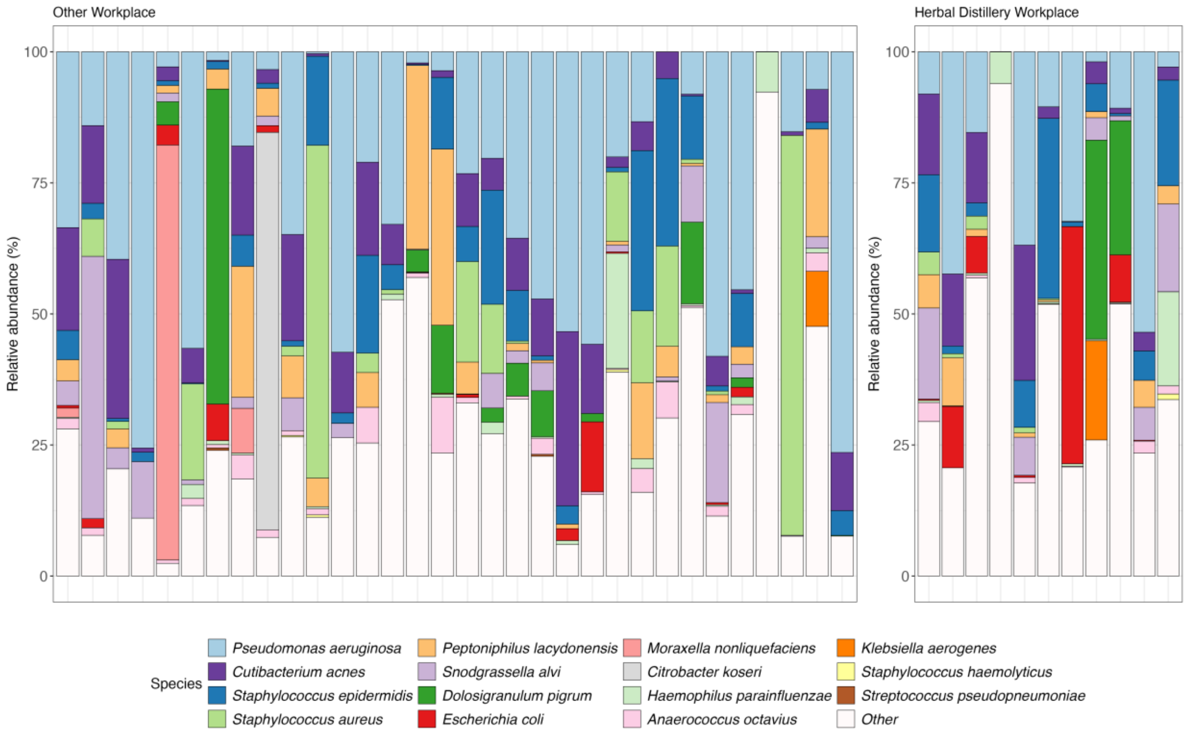

Supplementary Figure 1. The diversity of microbial species in the noses of healthy volunteers differentiated by the workplace: those working in herbal plant distilleries and those not associated with distilleries. Each color represents a different species, with a key for identification.

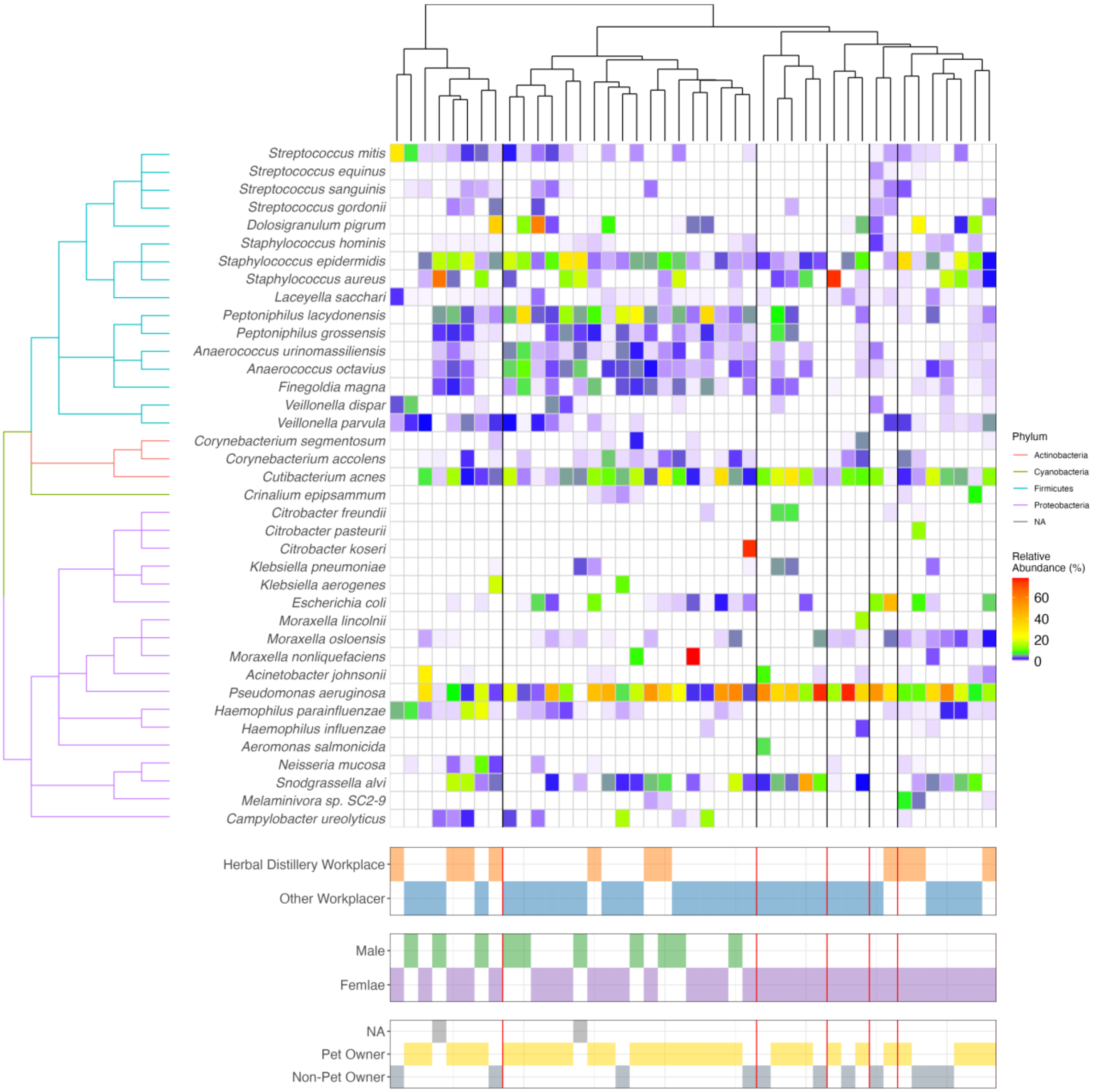

Supplement Figure 2. Nose Microbiome Diversity in Healthy Volunteers: This heat map illustrates the relative abundance of 38 bacterial species, chosen by selecting the twelve most abundant species from each sample across various samples. These species are organized into four phyla: *Proteobacteria*, *Firmicutes, Actinobacteria*, and *Cyanobacteria*. Each row represents a unique bacterial species, and each column indicates a distinct sample. After calculating the silhouette clustering index, we identified an optimal configuration with 6 clusters marked by black vertical lines. Below the heat map, plots display data on demographics (such as sex) and environmental factors (including pet ownership and workplace) for each sample; these plots outline cluster boundaries in red.

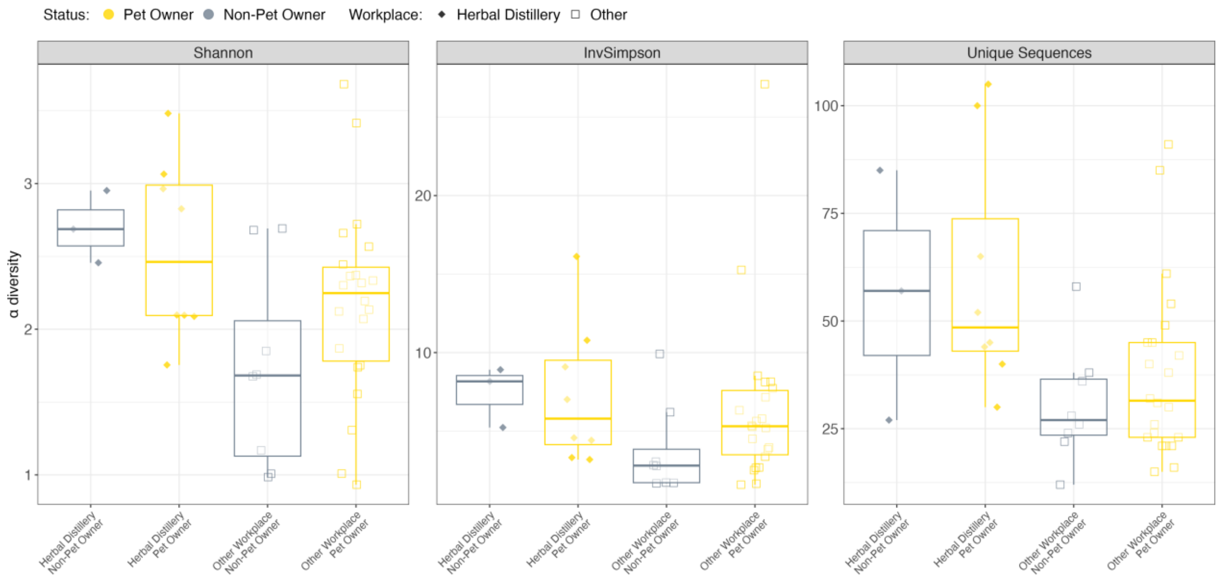

Supplement Figure 3. Boxplots illustrating the alpha diversity indices Shannon, Inverted Simpson, and Richness (number of unique identified taxonomies) across subgroups have been presented. These subgroups are distinguished by a combination of workplace characteristics (herbal distillery vs. other workplace) and pet ownership status.
